# Talent Cultivation of New Ventures by Seasonal Autoregressive Integrated Moving Average Back Propagation Under Deep Learning

**DOI:** 10.3389/fpsyg.2022.785301

**Published:** 2022-03-24

**Authors:** Fanshen Han, Chenxi Zhang, Delong Zhu, Fengrui Zhang

**Affiliations:** ^1^Graduate School, Gachon University, Seongnam, South Korea; ^2^School of Business, Gachon University, Seongnam, South Korea; ^3^School of Management Engineering, Anhui Institute of Information Technology, Wuhu, China; ^4^College of Life Science, Sichuan Agricultural University, Yaan, China

**Keywords:** deep learning, seasonal autoregressive integrated moving average back propagation, neural network, innovative talents, talent demand

## Abstract

This study combines the discovery methods and training of innovative talents, China’s requirements for improving talent training capabilities, and analyses the relationship between the number of professional enrollments in colleges and universities and the demand for skills in specific places. The research learns the characteristics and training models of innovative talents, deep learning (DL), neural networks, and related concepts of the seasonal difference Autoregressive Moving Average (ARMA) Model. These concepts are used to propose seasonal autoregressive integrated moving average back propagation (SARIMA-BP). Firstly, the SARIMA-BP artificially sets the weight parameter values and analyzes the model’s convergence speed, superiority, and versatility. Then, particle swarm optimization (PSO) algorithm is used to pre-process the model and test its independence. The accuracy of the model is checked to ensure its proper performance. Secondly, the model analyzes and predicts the relationship between the number of professional enrollments of 10 colleges and universities in a specific place and the talent demand of local related enterprises. Moreover, the established model is optimized and tested by wavelet denoising. Independent testing is done to ensure the best possible performance of the model. Finally, the weight value will not significantly affect the model’s versatility obtained by experiments. The prediction results of professional settings and corporate needs reveal that: there is a moderate correlation between professional locations and corporate needs; colleges and universities should train professional talents for local enterprises and eliminate the practical education concepts.

## Introduction

In the context of the requirements of an innovative country, innovating personnel training is the basis for China to become a country with innovative technologies, and it is also the only way to rejuvenate the country through science and education and strengthen the country with talents (Ge, 2020). In recent years, the lack of technology in cultivating innovative talents has severely hindered the implementation of China’s innovation strategy. There is still no effective solution to the problem of talent innovation, such as “the Qian’s Doubt” (Yang, 2021). The Chinese government is required to “improve first-class talent innovation capabilities” and “use high-tech to improve the speed of talent training reform” as China enters the stage of socialism with Chinese characteristics (Liu et al., 2019). With the emergence of new technologies, such as deep learning (DL), neural networks, and big data, applying them in the cultivation and excavation of innovative talents has become a hot research target of the new generation.

Artificial intelligence (AI) is an emerging technology, essentially using computing to connect with other devices to simulate how the human body thinks. Machines can achieve human functions through reasoning, knowledge, planning, learning, communication, perception, moving objects, using tools, and manipulating machinery. In talent training and collection, it is challenging for people to predict the results due to many objective factors. Therefore, AI technology is used as a way of talent training. In the past, technologies, such as neural networks and deep learning, were used in talent training, mostly from the perspective of enterprises. This can easily lead to the cultivation of talents in unrelated areas, and the final number of talents acquired is small, which cannot meet the needs of the company’s positions. This study conducts talent review and prediction from the professional types of colleges and universities in a certain area. The forecast result is a reference for local enterprises, enabling them to change their talent training standards to obtain more and higher-quality talent resources.

Artificial intelligence technology has a high degree of matching with talent training and collection. It is fully capable of this job. Therefore, AI and neural networks are being studied. The research learns a deep neural network (DNN) technology that combines DL and neural networks. After understanding related concepts and uses, a job prediction model is implemented using seasonal autoregressive integrated moving average back propagation (SARIMA-BP). The research uses the convergence rate to judge the reliability and usability of the model before conducting the research, collects the demand for relevant talents and the number of professional college enrollment in a certain place in recent years to predict the demand for positions, and then predict the demand in related fields. Talent demand matching trend, education level, and enterprise’s talent demand put forward relevant, constructive suggestions. The innovation is to combine DL and neural networks to establish a talent forecasting model. The correlation between talent demand and college specialty is used to determine the enrollment and enterprise needs to establish a strategy. This research can provide companies with strategies in recruiting requirements, help related companies quickly and accurately find talent resources that are highly compatible with professional needs, and expand the boundaries of the optimization and application of AI. There are multiple and even conflicting objectives in most public sector systems. Power factors often disturb these numerous objectives’ choice and weight ranking, and it is not easy to reach a consensus. The goals set by the public sector are often abstract and general. Public organizations with the same function have regional differences, and their scale and size are also different. It is unfair to measure their performance with the same performance indicators. The innovation of this study lies in the talent prediction based on talent resources and the optimization strategy for recruiting companies to change recruitment goals. Firstly, the Iceberg and Onion models illustrate the four elements of cultivating innovative talents starting from the characteristics and training methods of creative talents. Then, the DNN structure under the AI field is introduced from the single-level structure of the neural network and the memory unit components. The seasonal difference Autoregressive Moving Average (ARMA) model is proposed to combine with the established SARIMA-BP forecasting model. The convergence of the model and the matching degree of human resources are tested. The results of the research are drawn. Firstly, the cultivation mode of innovative talents, cultivation strategies, and social needs of innovative talents are described. Secondly, the technical concept of BP neural network (BPNN) is introduced, and the concept of SARIMA is introduced as the technical support for establishing the model. Thirdly, the SARIMA-BP prediction model is set to analyze the influencing factors of innovative talent cultivation. Moreover, the test method of wavelet denoising can independently test the model, including the model’s accuracy, various low-frequency signals, and error prediction. This method facilitates optimization and refinement of the results before they are studied. The PSO algorithm is used to verify the independent performance of the model. The experimental results show that the weight value will not significantly impact the model generality. The prediction results of specialty setting and enterprise demand show a moderate correlation between specialty setting and enterprise demand.

## Literature Review

The talent discovery ability of various universities has been improved with the wide application of big data technology. Qin et al. (2020) proposed the degree of matching of qualifications and job requirements, subjective influence, and low efficiency. They developed a topic-based ability to fit the neural network framework from the perceptron to the job to reduce the employer’s dependence on manual workers and provide better interpretable fitting results. This novel training mechanism addresses biased negative labels (Qin et al., 2020). Big data technology is used in talent training. Malah et al. (2020) improved the education services of talent and intellectual schools to train students who can enter the labor market and contribute to the country’s development. They created the Internet of Things to establish correct management strategies in the educational environment and create a comprehensive (safety, health, and economic) database that can be wholly relied upon (Malah et al., 2020). In addition, Gonzalez et al. (2019) researched the reliability, effectiveness, and fairness in the talent organization environment. AI and machine learning (AI/ML) applications are used for talent assessment and selection. They provided experimental evidence that AI and ML may cause adverse reactions to job applicants during the selection process and emphasized that psychologists, computer scientists, legal scholars, and members of other professional disciplines should strengthen cooperation (Gonzalez et al., 2019).

Many emerging technologies are used for talent training and mining. However, new neural network technology has not achieved much in talent mining. Therefore, neural network technology may play a unique role in talent mining. These documents show that many scholars are already exploring new strategies for the collection and training of human resources.

## Materials and Methods

### Characteristics of Innovative Talents

**Figure 1:**
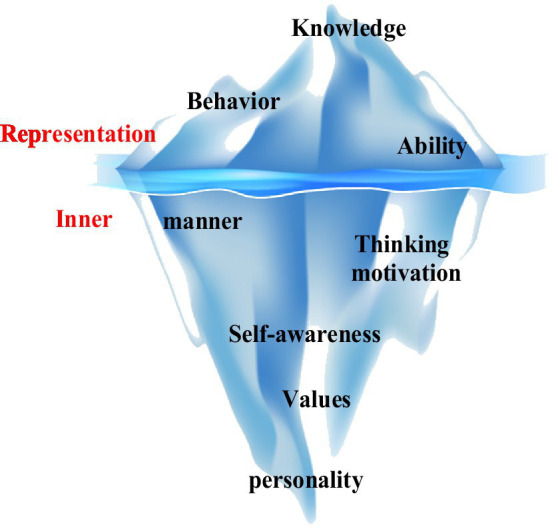
Iceberg model of talent characteristics.

Regarding the characteristics of innovative talents, Western scholars divide people’s creative morality into two types, explicit and implicit, based on the quality model theory. The iceberg model was established (Han and Zhang, 2021), as shown in Figure 1. The iceberg above the water symbolizes the ability of people to change their knowledge and ability. The iceberg below the surface of the water symbolizes the inner characteristics of people’s life concepts, moral quality, and human emotions that greatly influence the shaping of individuals. From the overall analysis, the difficulty of shaping the individual’s rate has increased. The influence of various factors on the personality has gradually increased from the top of the iceberg to the bottom.

**Figure 2:**
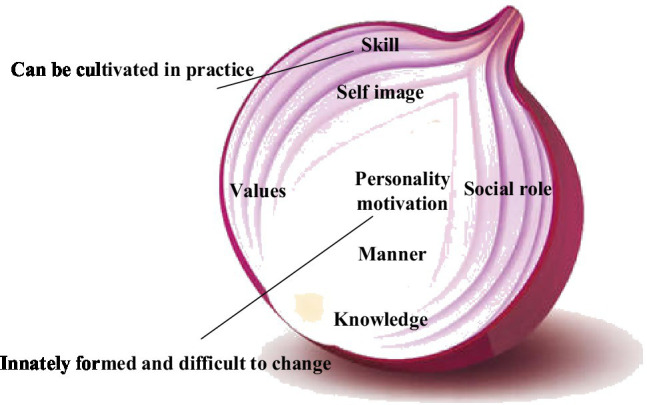
Onion model diagram of talent characteristics.

Subsequently, in Figure 2, the onion model is based on the iceberg model (Cao and Dong, 2021). The factors, such as knowledge and ability that are easy to change, are regarded as the epidermal part of the onion. The internal factors that are difficult to transform are considered the core part of the onion. Factors in the core part indicate that more effort is needed to make changes.

**Figure 3:**
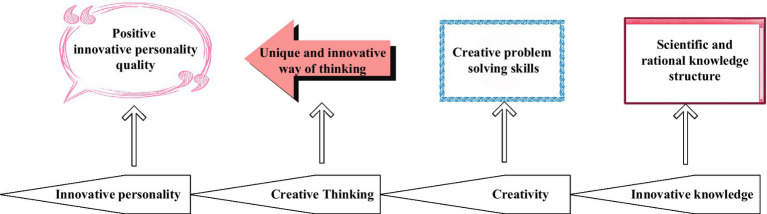
Four elements of personality shaping.

In Figures 1, 2, the research field summarizes the quality characteristics of innovative talents. Experts divide the process into four stages (Fetvadjiev and He, 2019) on two factors, the critical part of improving personal innovation ability and the leading role in personality shaping, as shown in Figure 3. Individuals must use reasonable and scientific knowledge to carry out innovations to solve problems encountered and cultivate their unique and innovative thinking mode to achieve an upright, positive, and imaginative personality.

**Figure 4:**
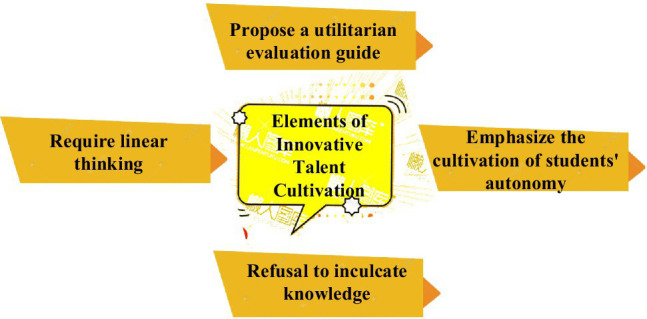
Four elements of talent training.

Appropriate technology is a basis for training talents to improve the discovery and training of innovative talents. Some experts use AI technology to divide the elements of talent training into colleges and universities (Ma, 2019), as shown in Figure 4. It is divided into four aspects: removing utilitarianism, abandoning linear thinking, refusing to instill knowledge forcibly, and cultivating students’ independent innovation.

Both the iceberg model and the elements of talent training show the importance of the social environment and personality elements to cultivate talents. But the most important thing is that colleges and universities should reasonably divide the proportion of majors according to the needs of majors and cannot abandon the original intention of education because of the practical nature of a major. Therefore, cultivating and exploring innovative talents needed by the market requires education managers to provide sufficient conditions for individuals. Universities need to adjust their professional proportions according to the current corporate talent demand model, and it is necessary to eliminate utilitarian educational concepts (Zhu, 2020).

### Deep Neural Network

**Figure 5:**
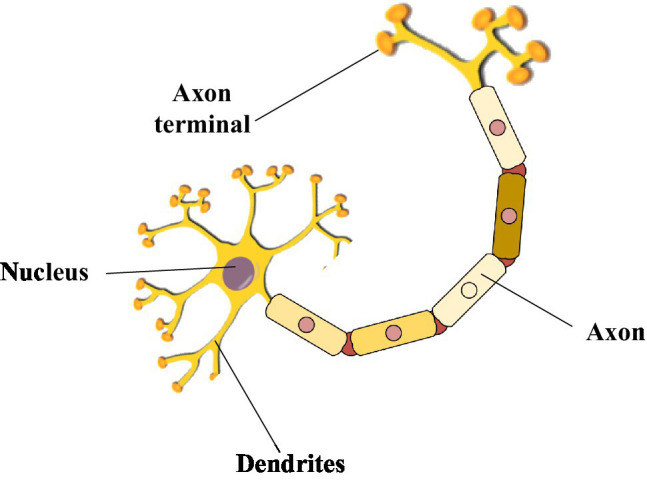
The simple neural reflex structure of the human body.

The hot research technology in the 1980s was artificial neural networks. The neural network structure is based on it by imitating the human brain’s neural structure in Figure 5 (Sun et al., 2021). The structure of a neural network is like a neural reflex unit in the human body. It touches the outside world through axons and transmits signals from nerves to cells in neurons. Therefore, research experts used computers to model and solve complex mathematical calculations (Hasson et al., 2020).

**Figure 6:**
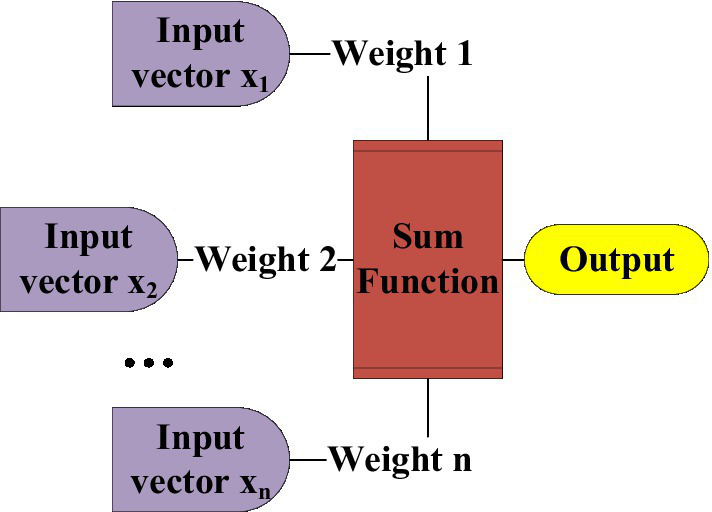
Single-layer structure of neuron unit.

The design of the neural network also refers to the neural network of the human body. The essence is to simplify the biological model. Similarly, the single neural reflex structure of the human body is represented by a scientific block diagram, which is the most basic single-layer neural network structure in the neural network (Indolia et al., 2018). Figure 6 is a single-layer simulation diagram of a single neuron unit. The single-layer simulation of a single neuron unit transmits the data information from the input layer to the neuron unit, processes the data through functional operations, and finally outputs the required data.

**Figure 7:**
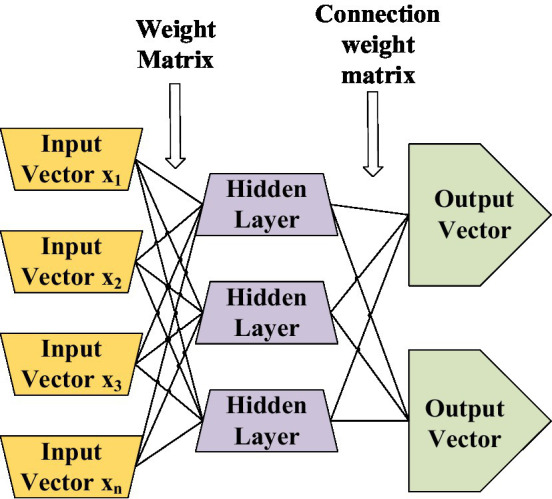
Multilayer neuron structure diagram.

The model built by adding new layers constitutes a multilayer neural network (Chou et al., 2019), as shown in Figure 7. With the increase of the layers, the multilayer neural network structure can further illustrate the characteristics of the data, and the simulation function has higher accuracy. The first layer takes the edge feature as the focal point, the second layer takes the edge composition shape as the focus point, and the third layer simulates the pattern feature composed of the shape as the focal point, as shown in Figure 7. More and more complex data can be processed, and feature differences can be made evident by dividing different items.

**Figure 8:**
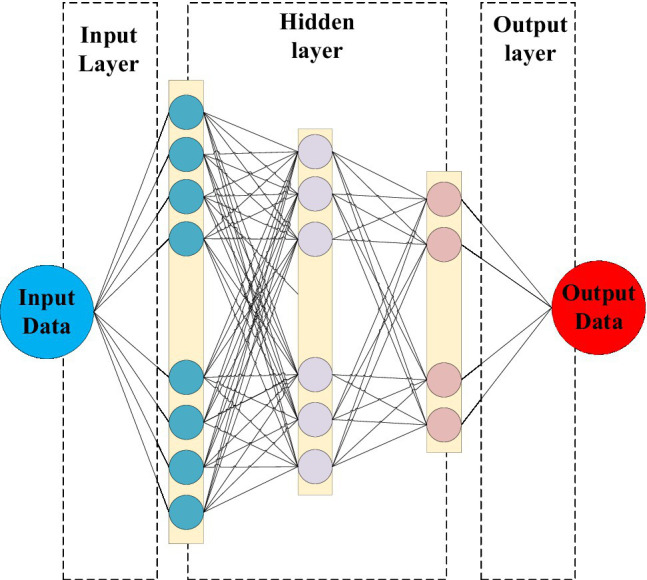
Deep neural network (DNN) structure.

With the development of the times, DNN has been proposed, a technology that can perform more complex calculations, as shown in Figure 8. DNNs can use more neural networks as sub-networks and build many hidden layers. And each independent hidden layer can calculate the output data of the previous layer, calculate and express complex data with multiple features, and get more and larger data sets than ordinary neural networks (Bianco et al., 2018).

DL combined with neural network AI technology has achieved outstanding results in recent years. For example, ImageNet won the game (Guo et al., 2019); in the Go game, AlphaGo won Lee Sedol (Bory, 2019). These results all show the rapid development of DNN technology.

**Figure 9:**
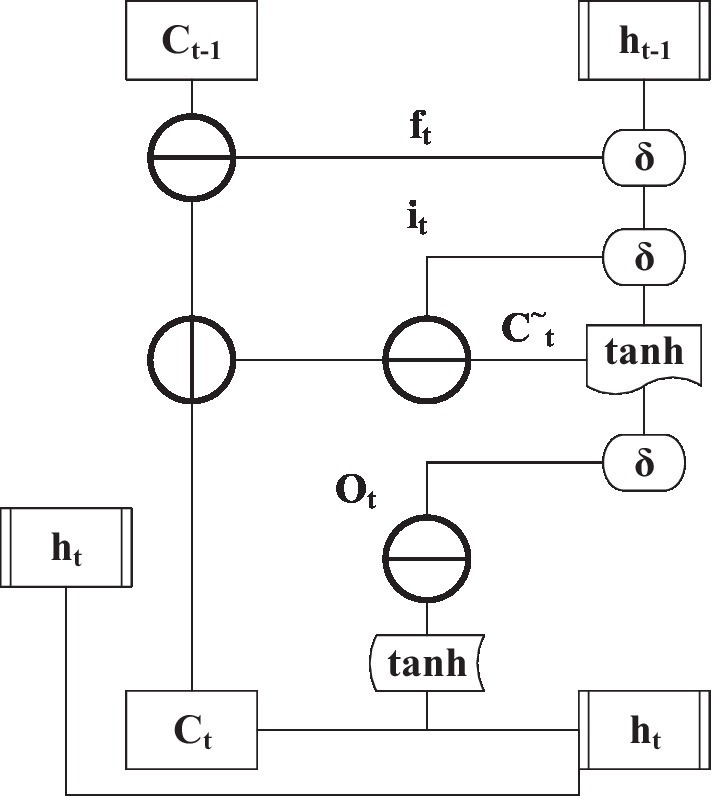
Structure of a single memory unit in a DNN.

In Figure 8, each layer of DNN is divided into many memory units. The structure of each memory unit is composed of different types of departments and processing functions, as shown in Figure 9.

Deep neural networks are different from ordinary neural networks. In addition to input and output gates, there are also forget gates. In Figure 9, *i_t_* is the input gate. *f_t_* is forgotten door, *O_t_* is the output gate, C^~^*_t_* is the current state of the input data after being processed by the tanh function, *C_t_* is a vector value, δ is a sign function, and *h_t_* is the output data of the neural unit. These gates can individually change and extract data and influence the results of the following component to achieve the purpose of independent learning and adaptation (Xu et al., 2018).

**Figure 10:**
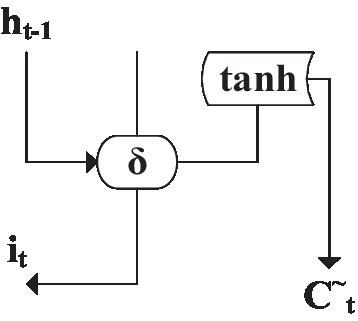
Memory unit input gate structure.

Figure 10 is the structure of the input gate (Tanaka, 2020), which updates valuable data by filtering useless information.

Trigger output *i_t_* and update output C^~^*_t_* can be solved by Equations (1) and (2):


(1) $$i_{t}=\delta \left(W_{\mathrm{xi}}x_{t}+W_{hi}h_{t-1}+b_{i}\right)$$

(2) $$C_{t}^{\sim }=\tanh\left(W_{xC}x_{t}+W_{hc}h_{t-1}+b_{c}\right)$$

Among them, $W_{x}$ is the weight value of the input data of the neural network. $W_{h}$ is the weight value of the t-th input data from the neuron. $h_{t-1}$ is the output data of the previous neuron. *b* is the amount of deviation corresponding to the neuron.

**Figure 11:**
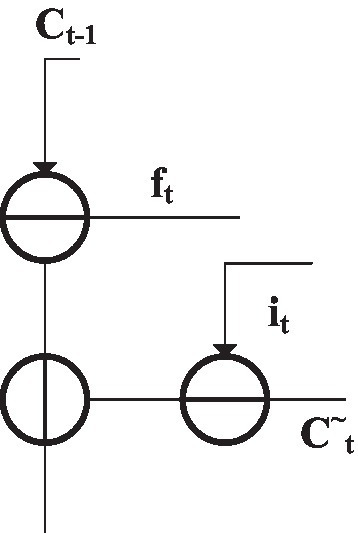
State layer structure of memory unit.

Figure 11 is the state neural layer structure (Chu et al., 2019), and the trigger output *C_t_* is solved by Equation (3):


(3) $$C_{t}=f_{t}C_{t-1}+i_{t}C_{t}^{\sim }$$

$C_{t-1}$ is the output data of the previous neural unit.

**Figure 12:**
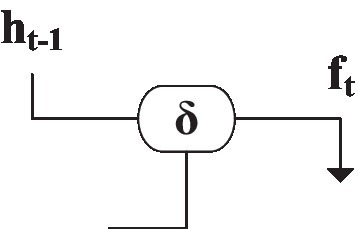
The structure of the forget gate of the memory unit.

Figure 12 is the structure of the forget gate (Chen et al., 2020). The data saved in the hidden layer can be selectively saved according to the sign function. *f_t_* is the trigger output, and the solution is shown in Figure 12:


(4) $$f_{t}=\delta \left(W_{x}x_{t}+W_{hf}h_{t-1}+b_{f}\right)$$

**Figure 13:**
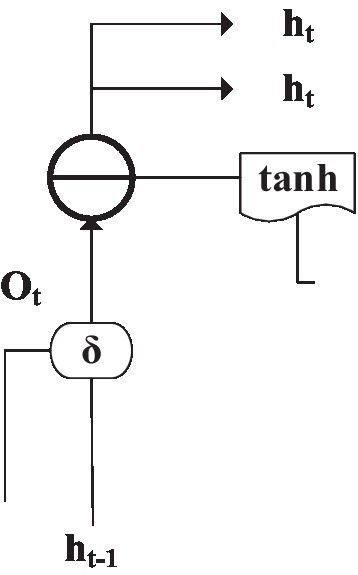
The structure of the memory unit output gate.

According to the sign function, the output gate (Vahora and Chauhan, 2019) can save the current output data in the hidden layer. In this way, the output of the hidden layer is controlled, and the trigger output *O_t_* is solved by an equation. The structure is shown in Figure 13:


(5) $$O_{t}=\delta \left(W_{\mathrm{xi}}x_{t}+W_{hi}h_{t-1}+b_{o}\right)$$

In Equation (5), $W_{\mathrm{xi}}$ and $W_{hi}$ are the weight values from the input layer to the corresponding gate. $W_{xc}$ and $W_{hc}$ are the weight values output by the module to the corresponding gate at the previous moment. $W_{xo}$ and $W_{ho}$ are the weight values from the previous cell neuron to the corresponding gate. b is the deviation of the corresponding door currently. The neural network output h_t_ can be obtained, as shown in Equation (6):


(6) $$h_{t}=o_{t}\tanh\left(C_{t}\right)$$

### Seasonal Autoregressive Integrated Moving Average

In the 1970s, the Autoregressive Integrated Moving Average model (ARIMA) was proposed by Western scholars. The ARIMA model is a solution to the problem of an unbalanced sequence (Hou et al., 2021). In addition, the ARIMA model is widely used in the financial, economic, agricultural, and meteorological industries (Awel, 2018; Bharati and Singh, 2019; Khedmati et al., 2020; Lai and Dzombak, 2020). The ARIMA model has also been used in the education industry, but the prediction model of the ARIMA model for the talent education industry is relatively rare.

In the ARIMA model, d is the order of difference, and the difference equation is Equation (7):


(7) $$\phi B\Delta^{d}x_{t}=\delta +\theta \left(B\right)u_{t}$$

The difference operator is:


(8) $$\Delta x_{t}=x_{t}-x_{t-1}=\left(1-B\right)x_{t}$$

However, the ARIMA model cannot perform low-level processing on some data with special dynamic laws (such as temperature, precipitation, electric energy load, and traffic, called seasonal series data). Therefore, SARIMA needs to improve the accuracy of the model.

Compared with the traditional ARIMA model, the essential difference of SARIMA is that it increases the expansion of the seasonal series (Farsi et al., 2021). Using period parameters, autoregressive parameters, difference, and average moving parameters can eliminate the influence of seasonal data. Time series (*Y_t_*, *t* = 1, 2...) have periodic and trend characteristics. Considering *Y_t_* as a non-stationary series with seasonal fluctuations but no obvious fluctuations, the seasonal difference in the first stage is expressed as Equation (9):


(9) $$\nabla_{s}Y_{t}=Y_{t}-Y_{t-s},\left(t>s\right)$$

The equation for the second stage is Equation (10):


(10) $$\nabla_{s}^{2}Y_{t}=\left(Y_{t}-Y_{t-s}\right)-\left(Y_{t-s}-Y_{t-2s}\right),\left(t>2s\right)$$

After that, *Y_t_* is subtracted *A* times and converted into a stationary series. *X_t_* is expressed as Equation (11):


(11) $$X_{t}=\nabla_{s}^{D}Y_{t},\left(t>D\right)$$

The model is Equation (12):


(12) $$\Phi \left(B\right){\left(1-B^{s}\right)}^{D}Y_{t}=\Theta_{Q}\left(B\right)\varepsilon_{t}$$

Among them:


(13) $$\Phi \left(B\right)=1-\varphi_{1}B^{s}-\varphi_{2}B^{2s}-\ldots -\varphi_{P}B^{Ps}$$

(14) $$\Theta_{Q}\left(B\right)=1-\theta_{1}B^{s}-\theta_{2}B^{2s}-\ldots -\theta_{P}B^{Ps}$$

$\varphi \left(B\right)$ represents the autoregressive polynomial characteristic of the sequence *Y_t_*. $\Phi \left(B\right)$ represents the seasonal autoregressive polynomial characteristic. $\theta \left(B\right)$ represents the seasonal average moving polynomial characteristic expression. $\Theta \left(B\right)$ represents the seasonal average moving polynomial characteristic. B represents the delay factor. P represents the seasonal regression parameter. $\varepsilon_{t}$ represents the residual, and the model is expressed as Equation (15):


(15) $$\varphi \left(B\right)\Phi \left(B\right){\left(1-B\right)}^{d}{\left(1-B^{s}\right)}^{D}Y_{t}=\theta \left(B\right)\Theta_{Q}\left(B\right)\varepsilon_{t}$$

In Equation (15), $\theta \left(B\right)$ and $\Theta_{Q}\left(B\right)$ represent the seasonal relationship in the sequence. $\varphi \left(B\right)$ and $\Phi \left(B\right)$ represent the quantitative relationship between neighbors. When SARIMA does not have seasonal characteristics, it degenerates into an ARIMA model.

### Implementation of SARIMA-BP Prediction Model

**Figure 14:**
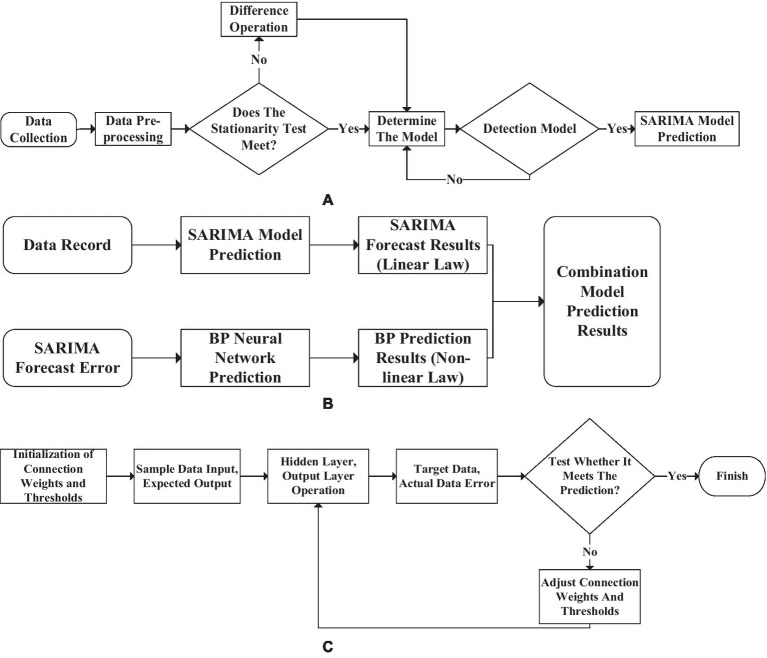
Seasonal autoregressive integrated moving average back propagation (SARIMA-BP) model data prediction process. [**(A)**: the SARIMA prediction model, **(B)**: the combined prediction model, **(C)**: the BPNN prediction model].

The talent needs of enterprises will be affected by many objective factors. Therefore, it is necessary to investigate and predict the matching degree of professional enrollment between enterprises in a certain place and local universities. The SARIMA, which can accurately reflect seasonal data, is combined with BP neural network for prediction (Jiang et al., 2017) to provide data support for local enterprise talent discovery work. BPNN generally has a high degree of non-linear performance and generalization strength, which makes more detailed data prediction through the characteristics of many iterations. Solving the main defect of slow convergence speed of neural network generally makes spatial analysis of neural network through GA to achieve better search space. Then BPNN combined with SARIMA technology is used to search for the optimal solution. The most outstanding feature of SARIMA is that it can accurately capture the periodic characteristics in the data flow to obtain better prediction data. Still, its defect is that the model cannot express the sequence change of data in the form of law. Therefore, it is necessary to summarize the change law as much as possible with the SARIMA model and then regularize the data with the mapping relationship in theory. The data stream is obtained through periodic difference processing to show randomness. After receiving the common law of the data, the difference in the data stream will become a random process. Hence, the data has a significant partial correlation. The forecast flow chart of the SARIMA-BP model is shown in Figure 14.

The essence of the prediction process of the combined model is to use two models to predict separately, synthesize the results and analyze. Finally, judge whether the prediction result meets expectations.

The work that needs to be done before predicting the result data is shown in Table 1.

**Table 1:** Work steps and content.

Work step sequence number	Specific content
Work step 1	First, the data are obtained and processed in advance, and incomplete data are repaired or eliminated.
Work step 2	Check the stability of the data model. If the data model is not stable, it needs to be smoothed by a different calculation.
Work step 3	Recognize the model by learning related graphics to determine the model parameters.
Work step 4	After the error analysis of the data and the parameters of the SARIMA model are determined, data prediction can be made. If the degree of convergence is in the proper position, it can be predicted by combining with BPNN.
Work step 5	SARIMA-BP model is used to predict the error. When the error is reached, the prediction is finished and the data is obtained.

**Figure 15:**
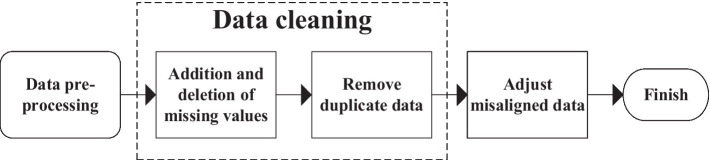
Data pre-processing flow.

Pre-processing of data requires analysis, repair, and transformation of some incomplete data. For data that cannot be repaired, it should be appropriately eliminated. The detailed pre-processing steps are shown in Figure 15. First, make appropriate repairs for some of the missing data in the database. If too much data is missing and cannot be restored, remove it based on the actual situation. Second, due to various objective factors of the model, the output data will be duplicated. Part of the duplicate data appears because of the similarity of the reference factors in the forecasting process. When there is too much same data due to model failure and other factors, the redundant data should be deleted.

Repair the data (Roy et al., 2018) and delete the missing incomplete or little relevant data. After data pre-processing, the correlation between the data is chaotic, so it is necessary to check the misalignment of the data. The data misalignment check repairs the error compensation problem caused by excluding the erroneous data in the data table and repeats the above work until complete and precise correct data is sorted out. The data are revised by investigating the relevant data of A students’ enrollment in the same major in 10 universities in A particular region from 2013 to 2020. After data correction, the data obtained are shown in Table 2.

**Table 2:** Number of students enrolled in a certain major in 10 universities in a certain place, 2013–2020.

Year	Total number of recruits in 10 colleges and universities	Number of A-Professional Enrollment	A proportion of professional enrollment in total enrollment
2013	45,797	8,408	18.35%
2014	44,518	9,422	21.16%
2015	44,754	9,580	21.40%
2016	46,109	9,835	21.32%
2017	47,241	9,898	20.95%
2018	40,836	9,932	24.32%
2019	49,334	10,085	20.44%
2020	50,112	10,334	20.62%

The convergence rate of the model is analyzed after determining the number of differences and pre-processing the data. Convergence speed is a judgment of the superiority of the model. It is necessary to observe the convergence speed of the algorithm in the model. Input the model for calculation simulation, and check the convergence speed of the algorithm in the model by artificially setting the weight value. The specific setting weight parameters are shown in Table 3.

**Table 3:** Weight setting values for convergence speed test.

	Data serial number									
Number	1	2	3	4	5	6	7	8	9	10
Weight value	80	79	78	77	76	75	74	73	72	71
	40	39	38	37	36	35	34	33	32	31
	20	21	22	23	24	25	26	27	28	29
	20	21	22	23	24	25	26	27	28	29

The weight is the actual value associated with each element, indicating the importance in predicting the final value. The weight associated with each function conveys the importance of that function in the predicted output value. Features close to zero are less important in the prediction process than features with larger weights. If the weight associated with a component is positive, there is a direct relationship between the feature and the target value. If the weight associated with the feature is negative, there is an inverse relationship between the feature and the target. Before performing principal component analysis on the data, the data must be standardized because the metrics of various types of data are different, such as calculating economic indicators. The value range of the output value of the tertiary industry in GDP is between 0 and 1.

The subjective experience method refers to how the evaluator assigns weights to each element based on the experience of an individual or a group of evaluators. The advantage of this method is that it is simple, convenient, and fast, and it can use the knowledge and experience accumulated by the evaluator for a long time. The evaluator can make rapid adjustments according to the actual situation and changes in the environment, with substantial flexibility and pertinence. But its drawbacks are also very prominent. This method is based on the experience of individuals or a small number of people. This method has great limitations and instability due to personal knowledge, range of activities, cognitive ability, and personality preferences. Especially when the evaluator and the evaluated person have an interest or conflict, the determination of its weight will often deviate from objective standards, and even corrupt behavior will occur. Therefore, in modern quality management, this method is often used as a preliminary survey or evaluation design, and the final weight determination must be tested or adjusted by other methods.

The unit weighting method refers to the practice of directly adding the original evaluation data of each element to obtain the evaluation result, as shown in Equation (16):


(16) $$X_{c}=x_{1}+x_{2}+\ldots +x_{n}$$

$X_{c}$ is the total evaluation result (score). x_1_, x_2_, ..., x*_n_* are the original evaluation data (or scores) of each sub-element, respectively. On the surface, this method puts the sub-elements in an equal position, but it is not. Because different sub-elements use different tools for evaluation, their dimensions (units) are also different. It is meaningless to add data in different units directly, even if the dimensions of each sub-element are the same under certain conditions. In this method, the score of each sub-element is weighted in proportion to its standard deviation. This weighting method has been used many times in Chinese exams, especially when combining low-level data. This method is used in the examination by dividing the test questions into test paper points.

The weighted average method refers to weighting the sub-elements based on the ratio of the scores of the sub-elements in the population. Assuming that there are n sub-elements, and the evaluation data are X_1_, X_2_, ..., X*_n_*, the weight coefficient of each sub-element is calculated as Equation (17):


(17) $$Y=b_{1}x_{1}+b_{2}x_{2}+\ldots +b_{i}x_{i}$$

*b_i_* is the weight of the *i*-th element. *x_i_* is the evaluation raw data of the *i*-th element and the sum of the original data of the element evaluation. This weighting method is primarily used in simple assessment and group assessment. If the average of each sub-group is known, find the total average of the group.

The equal weighting method is a weighting method that assigns the same weight to each evaluation element. Due to the different evaluation tools and methods adopted by each evaluation element, the dimensions of the evaluation data and the distribution may also be different. Simply adding directly (such as unit weighting) is not equal weighting. Equal weighting is first to convert the scores of each evaluation element with additional units to a unified dimension with the same team and then add them to get the total score. The most used conversion scores are standard scores (*z*-scores or *T*-scores). That is, the scores of each evaluation element are first converted into standard scores, and then the standard scores of each component are added to form a total score *Z_c_*, as shown in Equation (18):


(18) $$z_{c}=z_{1}+z_{2}+\ldots +z_{n}$$

Z_1_, Z_2_, ..., Z*_n_* are the standard scores of each evaluation element. The condition for using equal weighting is: each part must have the same importance to the whole.

The matrix operation method is a method of calculating the weight vector by the matrix inverse operation method. The matrix algorithm assumes that when the number of people participating in the evaluation reaches a specific number, whether the statistical average of a certain index or the statistical average of the comprehensive evaluation value of experts regardless of the item, it can reach a satisfactory degree. The practice of foreign talent evaluation shows that this hypothesis is valid when the number of experts reaches more than 1,000. If there are *N* indicators in the indicator system, the evaluator will evaluate the *n* evaluators. The evaluation is carried out in two ways at the same time, one is the evaluation of sub-items, and the other is the overall evaluation. Thus, the total evaluation values of n evaluators are obtained: B_1_, B_2_, ..., B*_n_*. Meanwhile, *n* groups of evaluation values on each sub-item of each evaluation object are also obtained.

In this way, the required weight vector (A_1_, A_2_, ..., A*_n_*) is obtained. This method can largely eliminate the influence of accidental factors in the process of determining the index weight coefficient and is relatively stable. The disadvantage is that it requires too much manpower and material resources. The current application range is very limited. However, with the popularity of the Internet, this method will become more and more popular when the evaluation data is easier to obtain.

The relationship between the predicted results and the data is analyzed using the correlation coefficient method (Jiang et al., 2019). If X and Y increase or decrease for two sets of data (X, Y), the two sets of data have a positive correlation, and the correlation coefficient interval is [0, 1]. If X increases, Y shrinks or vice versa, then X and Y are said to be a negative correlation, and the coefficient interval is [−1, 0]. The closer the absolute value of the correlation coefficient to 1, the stronger the relationship. The variances of X and Y are represented by *σ_x_* and *σ_y_*, respectively, and cov (X, Y) is used to represent the covariance of X and Y. The correlation coefficient is calculated as Equation (16):


(19) $$\rho_{xy}=\frac{\mathrm{cov}\left(X,Y\right)}{\sigma x\cdot \sigma y}$$

The observation values studied are *X* = (x_1_, x_2_, …, x*_n_*), *Y* = (y_1_, y_2_, …, y*_n_*). Then, the sample coefficient is calculated as Equation (17):


(20) $$r_{xy}=\frac{n\sum x_{n}y_{n}-\sum x_{n}\sum y_{n}}{\sqrt{n\sum x_{n}^{2}-{\left(\sum x_{n}\right)}^{2}}\cdot \sqrt{n\sum y_{n}^{2}-{\left(\sum y_{n}\right)}^{2}}}$$

Using Equation (17), the relationship coefficient between X and Y in the data can be calculated, and the degree of the relationship can be analyzed.

### SARIMA-BP Model Pre-processing

**Figure 16:**
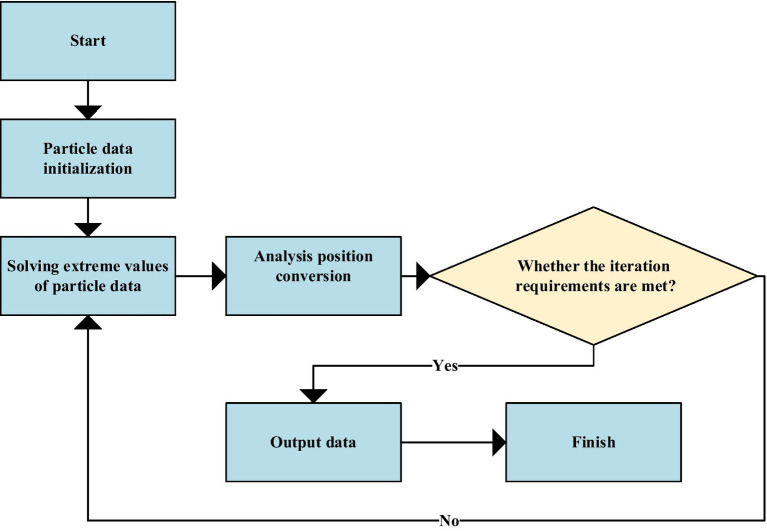
Pre-processing flow of SARIMA-BP model data.

Particle swarm optimization is an evolutionary computing technology, also known as particle swarm algorithm. It is an optimal search evolutionary algorithm based on group cooperation developed by simulating the foraging behavior of biological populations. The basic idea of PSO is to seek the optimal global solution through mutual cooperation and information exchange among all individuals in the population. The advantage is that the implementation is relatively simple and easy to implement and does not require any adjustment parameters. With the deepening of research in this area, the PSO algorithm has been widely used in parameters optimization of neural network models and support vector machines (SVM), weight optimization of combined models, etc., and has shown promising results. Figure 16 is a flow chart of data pre-processing for the SARIMA-BP model using the PSO algorithm.

In 1969, the idea of combinatorial predictive models was pointed out. The combined prediction model can significantly improve the prediction effect through the effective combination of single prediction models. People have carried out in-depth and extensive research in theory and evidence-based on this new way of thinking. The results show that due to the different reflection angles of the individual prediction models to the original data and the different proportions of the extracted information, the combined model incorporates the individual models. This concentrates more modeling skills, which can significantly reduce the systematic error of the prediction and can obtain a prediction value with higher accuracy than the single-term model. Compared with the idea of constructing a complex single-item model, the combined model recognizes the difficulty of building a real model and places more emphasis on fully including single-item models that reflect different pieces of information. The combined model is less sensitive to the model assumptions of the single-term model and has the characteristics of less risk of extreme prediction errors. Now, this method has become a research hotspot in many research fields.

Commonly used combination prediction methods include linear combination prediction model, optimal linear combination model, Bayesian combination model, and transfer function combination model. The application of a general linear combination forecasting model to time series is shown in Equation (21):


(21) $$y_{t}=w_{1}y_{t1}+w_{2}y_{t2}+\ldots +w_{n}y_{tn}$$

*y_t_* is the model prediction processing result with *t* as the period. *y_tn_*, respectively, represent *n* different prediction results, and the *w_n_* is the corresponding weight. The difficulty of the combination model lies in determining the optimal combination weight. If the weights are unreasonable, the prediction effect of the combined model may be worse than that of the single model. There are two weight determination methods: non-optimization methods and mathematical optimization methods.

Non-optimized methods mainly include the arithmetic mean method and common method.

#### Arithmetic Means Method

This method treats all models equally. That is, the weight coefficients of the single-item models are equal. The optimal number of weight values is obtained through the error objective function. Here, *n* different models are generally used for prediction, so that w_1_, w_2_, ..., w*_n_* are used as weight groups, and e_1_, e_2_, ..., e*_n_* are used as prediction errors, which satisfies Equation (22):


(22) $$w_{1}+w_{2}+\ldots +w_{n}=1,w_{i}\geq 1,i=1,2,\ldots ,n$$

By choosing an error objective function Q, the problem can be transformed into Equation (23):


(23) $$\mathrm{minQ}=f\left(e_{1},e_{2},\ldots ,e_{n}\right)$$

(24) $$w_{1}+w_{2}+\ldots +w_{n}=1$$

The optimal weights can be obtained by solving the optimization problem. Generally, the sum of squares of errors is taken as the error objective function.

Intelligent algorithms have advantages in parameter optimization, and using them for weight finding is a worthwhile choice. Suppose the error objective function value in the mathematical optimization method is used as the fitness value in the particle swarm optimization method. In that case, the weight coefficient in the combined model is obtained, and then the weight is substituted into the combined prediction model based on PSO.

For time series forecasting, an optimal combined forecasting model based on SARIMA and BPNN is proposed to fully use the advantages of the SARIMA model and BPNN model. The model combines the SARIMA model and BPNN and uses PSO to select the weight of the integrated model based on single-item model prediction. Specific steps are as follows:

1. SARIMA and BPNN models are used to forecast time series.
2. The residuals of each model are calculated to obtain the error objective function with the error sum of squares as the objective.
3. PSO is used to calculate the weights of each model. Substitute the obtained weight coefficients into the combination model to construct the optimal combination prediction model.
4. The combined model is used to make predictions compared with the single-term and non-optimal combined models.

The model is pre-processed and independently tested through the above steps, and specific performance verification is carried out in the following link.

### Wavelet Denoising of SARIMA-BP Model

Different forecasting methods can be used for the same problem in the field of forecasting. However, the useful information captured by each method is different due to the different modeling mechanisms of each different forecasting method. In this case, if the method with a larger prediction error is simply discarded, some useful information may be lost. This practice is unscientific, a waste of information, and should be avoided. In order to make full use of useful information and improve forecasting accuracy, a more scientific approach is to appropriately combine different forecasting methods to form a combined forecasting method.

In forecasting practice, combined forecasting models have been widely studied. Especially in the past two decades, prediction models combining different methods have been widely used. The primary purpose of the combination is to comprehensively utilize the valuable information provided by various methods to improve the prediction accuracy as much as possible. The linear combination method is generally used in the combination process.

A combined prediction model is proposed based on wavelet denoising, which combines cumulative autoregression with seasonal term, moving average, and BPNN. In this cooperative prediction model, wavelet denoising is used to remove the noise of the original data. Then, the low-frequency signals after wavelet denoising are modeled by cumulative autoregressive and moving average models and BPNN models with seasonal items, respectively. The corresponding model tests and predictions are made. Finally, variance and covariance methods combine the results predicted by the two individual models. Electricity load data is a time series with high noise due to the influence of the fluctuating electricity market. Direct prediction of noisy load data will result in higher errors. Wavelet transform is essentially an integral transform between different parameter spaces. Wavelet basis is the core of wavelet transform. Different wavelet bases have different effects on wavelet transform. The choice of wavelet base should consider different application fields and choose different ones. Different choices of wavelet bases play a key role in wavelet transformation. Corresponding wavelet functions are selected according to the specific situation that arises. Only in this way can the processing effect be better.

In power load forecasting, many different methods have been proposed. In order to deal with the load data with higher noise, a cumulative autoregressive and moving average and BPNN with seasonal term based on wavelet denoising is proposed. The forecasting model is used to make actual power load forecasting. Unlike other combined models, the prediction process of this integrated model is:

1. The wavelet denoising method is used to remove the noise of the original load data. In denoising with wavelet, wavelet transform decomposes the original data signal into low-frequency and high-frequency signals. Low-frequency signals give the characteristics of the original signal, while high-frequency signals are associated with noise. The high-frequency signal is removed, and the signal’s basic characteristics can still be preserved, thus eliminating the noise in the data.
2. SARIMA and BPNN models are used to model low-frequency signals, and the corresponding model tests and predictions are made.
3. A combined model based on the variance and covariance method to determine the combined weights makes a new combined prediction for the results predicted by a single model.

The corresponding absolute and relative errors can also be displayed by comparing the predicted and actual values of the proposed combined forecasting model. This integrated model can well capture the characteristics of the power load data, and the relative error fluctuates within a small interval near zero. This shows that the combined model has good statistical performance and high prediction accuracy.

## Results

### Model Convergence Rate

**Figure 17:**
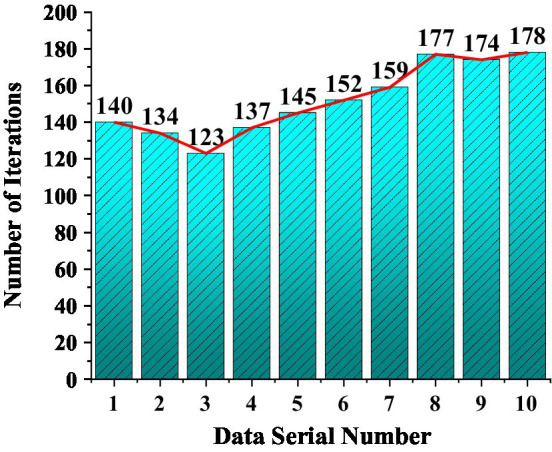
The relationship between the weight value and the number of iterations shows the convergence speed.

The superiority and versatility of the model are analyzed to explore the convergence speed of the SARIMA-BP. The research inputs the artificially set weight values in the method for simulation operation and draws the relationship data between the function weight values and the number of iterations into a statistical graph. The result is shown in Figure 17.

Figure 16 shows the influence of different weight values on the convergence speed of the model algorithm. The fastest model convergence rate is the third group, and the convergence rate is 123 s. The slowest convergence is the tenth group, which takes 178 s. The weight value of the third group is 78, 38, 22, 22. The weight value of the tenth group is 71, 31, 29, 29. The value of the weight has a negligible influence on the convergence speed of the model. The lower the number of iterations, the faster the convergence of the model, and the smaller the initial weight value. The more iterations of the model, the slower the convergence speed. The post-weight value has no obvious influence on the convergence speed, and the fastest convergence speed is the third group. The maximum or minimum value of the weight value will not affect the convergence speed of the model, and the weight value should be set according to the model itself.

### Matching of Specialty and Enterprise

**Figure 18:**
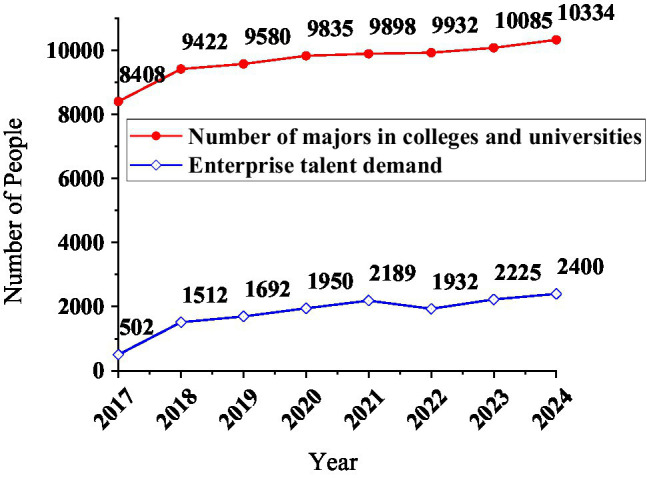
The curve of the number of professional enrollments in colleges and universities and the demand for talents in enterprises.

The number of persons set up for major A is obtained from 2013 to 2020, according to the data in Table 2. Use *X* to represent the number of students enrolled in the same major in 10 colleges and universities in a certain place, *X* = [8,408, 9,422, 9,580, 9,835, 9,898, 9,932, 10,085, 10,334]. Colleges and universities carry out a 4-year training plan for each student. Use the SARIMA-BP to predict the demand for talents, and the number of demands is represented by *Y*, *Y* = [502, 1,512, 1,692, 1,950, 2,189, 1,932, 2,225, 2,400]. Use drawing software to draw a diagram of the relationship between the number of professional college enrollments and the demand for talents in related enterprises, as shown in Figure 18.

The number of high-efficiency enrollment does not increase year by year. When drawing the image, the number of college professional enrollment parameters of the abscissa axis is arranged in ascending order. The number of professional enrollments in colleges and the demand for corporate talents in the corresponding year: the degree of deflection of the two curves is similar, indicating that the two have a clear correlation. Bring the X and Y data into the correlation coefficient calculation equation, and *r_xy_* = 0.69. Therefore, there is a certain relationship between the number of professional enrollments in colleges and universities and the demand for talents in enterprises, but not close.

## Discussion

This study uses the characteristic elements and training method requirements of innovative talents. It uses the iceberg and onion model as the theoretical basis to explain the performance of talent needs. Then DNN in the AI field is used as technical support, introducing the hierarchical structure and memory unit functions of neural networks. The convergence performance of the model and the matching degree of human resources are tested and combined with the seasonal difference ARMA Model to form a SARIMA-BP prediction model. The convergence performance of the model is stable, and after being predicted, there is a high degree of matching between the enterprise’s professional needs and the nature of talents. After independent testing and model pre-processing, it can ensure the normal operation and work of the model and ensure that the model can be used normally in experiments without interference from other objective factors. This is consistent with the research results of Preckel et al. (2020). They provided a general talent development framework suitable for a wide range of achievement fields through the talent development framework in achievement fields. Empirical research supported it by focusing on measurable psychological structure and its significance at different talent development levels and commented on the applicability of the support framework to build a model in specific fields (Preckel et al., 2020). Similar to the conclusion of this study, it is found that different factors impact the training mode of innovative talents. This solves the problem of low numbers and low quality of recruited talents caused by the mismatch of skills in enterprises’ current career recruitment process. The applicability of the model is further proved.

## Conclusion

In the context of China’s policy of an innovative and powerful country and the cultivation of new talents, the research studies the relationship between the number of students enrolled in a university’s major and the demand for talents from local related companies. Firstly, it summarizes the characteristics of innovative talents and the training methods of innovative talents. Secondly, the related concepts and algorithms of DL, BPNN, SSARIMA, and SARIMA-BP using DL are learned. By artificially setting parameters, the convergence calculation speed of SARIMA-BP is analyzed, and the conclusion of the superiority and versatility of the model is obtained. It is critical to select an appropriate single-item forecasting model based on the original data features in the linear combination forecasting model. The weight selection of the combined model is also crucial, and intelligent algorithms, such as PSO, effectively optimize the weights. Only when these aspects of work are done well can the established model achieve the desired effect. Afterward, SARIMA-BP is used to investigate the number of students enrolled in a particular major in 10 colleges and universities in a certain place from 2013 to 2020. The model is used to predict the enterprise talent demand in 4 years. The wavelet denoising method ensures the model’s prediction performance accuracy and optimizes the errors and residuals caused by objective factors during the model establishment process. The correlation coefficient equation calculates the close degree of the relationship between the two. The result obtained is that the two have a moderate degree of relationship. Therefore, it is held that (1) The city needs corresponding national policy support to provide professional education for local enterprises. (2) Eliminate unnecessary utilitarian education concepts, attach importance to the training of professional students, and discover and train professional talents for the needs of related enterprises and the country. The limitation is that it analyzes the relationship between the convergence speed of SARIMA-BP and the enrollment company but does not discuss how to improve the convergence speed and how to strengthen the cooperation relationship between universities and enterprises. This will be the focus of follow-up work.

## Data Availability Statement

The raw data supporting the conclusions of this article will be made available by the authors, without undue reservation.

## Ethics Statement

The studies involving human participants were reviewed and approved by Gachon University Ethics Committee. The patients/participants provided their written informed consent to participate in this study. Written informed consent was obtained from the individual(s) for the publication of any potentially identifiable images or data included in this article.

## Author Contributions

All authors listed have made a substantial, direct, and intellectual contribution to the work, and approved it for publication.

## Conflict of Interest

The authors declare that the research was conducted in the absence of any commercial or financial relationships that could be construed as a potential conflict of interest.

## Publisher’s Note

All claims expressed in this article are solely those of the authors and do not necessarily represent those of their affiliated organizations, or those of the publisher, the editors and the reviewers. Any product that may be evaluated in this article, or claim that may be made by its manufacturer, is not guaranteed or endorsed by the publisher.
